# Mild Systemic Oxidative Stress in the Subclinical Stage of Alzheimer's Disease

**DOI:** 10.1155/2013/609019

**Published:** 2013-12-18

**Authors:** Leandro Giavarotti, Karin A. Simon, Ligia A. Azzalis, Fernando L. A. Fonseca, Alessandra F. Lima, Maria C. V. Freitas, Milena K. C. Brunialti, Reinaldo Salomão, Alcione A. V. S. Moscardi, Maria B. M. M. Montaño, Luiz R. Ramos, Virgínia B. C. Junqueira

**Affiliations:** ^1^Instituto de Química, USP, Cidade Universitária, Avenida Lineu Prestes 748, 05508-900 São Paulo, SP, Brazil; ^2^Instituto de Ciências Ambientais, Química e Farmacêuticas, UNIFESP, Rua Arthur Riedel 275, 09972-270 Diadema, SP, Brazil; ^3^Departamento de Hematologia e Oncologia, FMABC, Avenida Príncipe de Gales 821, 09060-650 Santo André, SP, Brazil; ^4^Departamento de Medicina, UNIFESP, Rua Sena Madureira 1500, 04021-001 São Paulo, SP, Brazil; ^5^Departamento de Medicina Preventiva, UNIFESP, Rua Sena Madureira 1500, 04021-001 São Paulo, SP, Brazil

## Abstract

Alzheimer's disease (AD) is a late-onset, progressive degenerative disorder that affects mainly the judgment, emotional stability, and memory domains. AD is the outcome of a complex interaction among several factors which are not fully understood yet; nevertheless, it is clear that oxidative stress and inflammatory pathways are among these factors. 65 elderly subjects (42 cognitively intact and 23 with probable Alzheimer's disease) were selected for this study. We evaluated erythrocyte activities of superoxide dismutase, catalase, and glutathione peroxidase as well as plasma levels of total glutathione, **α**-tocopherol, **β**-carotene, lycopene, and coenzyme Q10. These antioxidant parameters were confronted with plasmatic levels of protein and lipid oxidation products. Additionally, we measured basal expression of monocyte HLA-DR and CD-11b, as well as monocyte production of cytokines IL1-**α**, IL-6, and TNF-**α**. AD patients presented lower plasmatic levels of **α**-tocopherol when compared to control ones and also higher basal monocyte HLA-DR expression associated with higher IL-1**α** production when stimulated by LPS. These findings support the inflammatory theory of AD and point out that this disease is associated with a higher basal activation of circulating monocytes that may be a result of **α**-tocopherol stock depletion.

## 1. Introduction

Alzheimer's disease (AD) is a neurodegenerative disorder resulting in progressive neuronal death and memory loss [1]. Histopathologically, the disease is characterized by intraneuronal neurofibrillary tangles (NFT) (aggregations of a hyperphosphorylated form of the microtubule-associated protein tau) and extracellular deposits of neuritic plaques composed of amyloid-**β**(A**β**) protein, a 40–43 amino acid proteolytic fragment derived from the amyloid precursor protein [2].

Although AD is considered a neurodegenerative disease, both inflammation and oxidative stress are present early and throughout its pathogenesis. One of the early clues as to AD as an inflammatory process was the finding that rheumatoid arthritis patients taking high levels of anti-inflammatory drugs had a low incidence of AD [3]. Inflammatory mechanisms may significantly contribute to disease progression and chronicity, with proinflammatory cytokines (notably IL-1*α* and IL-1*β*) being found throughout the brain of individuals with AD when analyzed at autopsy [1]. Many neuroinflammatory mediators including complement activators and inhibitors, chemokines, cytokines, reactive species, and inflammatory enzymes are generated and released by microglia, astrocytes, and neurons [4].

However, the precise implications of the inflammatory response for neurodegeneration have not been elucidated. A current hypothesis considers that an extracellular insult to neurons could trigger the production of inflammatory cytokines by astrocytes and microglia. The cytokines, namely, IL-1*β*, TNF-*α*, and IL-6, could affect the normal behavior of neuronal cells. Therefore, dysfunction at this core level may lead to abnormalities such as neurofibrillary degeneration in AD [5].

It is well characterized that there is oxidative stress in the AD brain. Lower plasma antioxidant levels and alterations in antioxidant enzyme activities were reported in mild cognitive impairment (MCI) patients and patients at early AD stages, suggesting an imbalance between reactive species production and antioxidant defense systems in the plasma of AD patients. This was substantiated by an increase in DNA, lipid, and protein oxidation products found in blood and cerebrospinal fluid (CSF) obtained from AD patients in comparison with controls [6].

At the same time microglial cells activation is prominent during AD. Microglia are bone marrow-derived cells of the monocyte lineages that, like peripheral tissue macrophages, become phagocytic and produce reactive oxygen species [3]. However, the evaluation of microglia activity during the development of AD in human patients is impossible due to its localization, thus justifying demand for models that use parameters of easy access and reflecting the state of activation of microglia. However, the studies with monocytes could contribute to the understanding of inflammatory component in AD, establishing, at the same time, the possibility of using a minimally invasive technique, such as blood collection.

As exposed, AD has an important component that involves inflammatory reactions associated with reactive species release and lower antioxidant levels. Thus, this paper aims at verifying blood oxidative markers and monocyte inflammatory parameters in elderly subjects who present with very mild cognitive impairment.

## 2. Methods

### 2.1. Subjects

Patients participating in this study were part of the last wave of Epidoso Project receiving monitoring on aging by a multidisciplinary team [7].

65 elderly subjects (mean age 82 years) were selected for this study. All patients had their functional capacity and laboratory profile evaluated. These seniors were initially subjected to a clinical survey conducted by a geriatrician, consisting of history taking, physical examination, and evaluation of mental autonomy, through the degree of cognitive function. Patients with a history of cardiovascular disease, cancer, or chronic inflammatory diseases as well as individuals with current inflammatory alterations, assessed by erythrocyte sedimentation rate or plasma levels of C-reactive protein, were not included in this study.

The study protocol was submitted to the Ethics Committee of UNIFESP and was approved under number 0859/03. All patients or their guardians signed informed consent.

### 2.2. Assessment of Patients' Cognitive Ability

Minimental state examination was used for screening cognitive deficits among patients [8]. The maximum score is 30 and scores lower than 24 were considered indicative of some kind of cognitive dysfunction.

Patients who scored less than 24 points in MMSE were then tested with CDR (Clinical Dementia Rating) [9, 10].

Patients were considered cognitively intact (INT group; *n* = 42) when CDR was <1, while others were included in the group of patients with probable Alzheimer's disease (AD group; *n* = 23) if CDR was ≥1.

### 2.3. Measurements of Erythrocytes Parameters

Blood samples from participants were obtained after 12 hours of fasting. Part of whole blood was used for flow cytometry. After plasma separation, erythrocytes were used to measure total glutathione content (TGSH) [11] and the remaining hemolysate to assess the activities of antioxidant enzymes [7], Cu-Zn superoxide dismutase (SOD), catalase (CAT), and glutathione peroxidase (GPx), all adjusted by hemoglobin concentration.

### 2.4. Measurements of Plasma Parameters

Plasma samples were stored at −80°C for later determination of *α*-tocopherol, *β*-carotene, lycopene, coenzyme Q10, and vitamin C [7]. For extraction, 20 *μ*L plasma aliquots were mixed with 100 *μ*L extraction solvent (50% ethanol proanalysis (pa), 50% 1-butanol pa, containing 5 mg butylhydroxytoluene/mL, and 8 *μ*mol Tocol/L as an internal standard) to extract the vitamins and to denature plasma proteins. The sample was then mixed by vortex for 30 s and centrifuged at 21 000 ×g for 10 min at 4°C. The supernatant was transferred to a polypropylene vial. This method uses precolumn and reverse phase C18 column and UV-VIS detector with deuterium lamp, with a mobile phase consisting of 80% acetonitrile, 3% methanol (100 mM ammonium acetate, 0.1% triethylamine), and 15% dioxan. The mobile phase is pumped through the system by a flow of 1.5 mL/min with maximum pressure of 350 Kgf and the minimum of 0. The wavelength was established by the method for each parameter and run time for it was 4 to 6 minutes.

Plasma was also used to determine the concentration of thiobarbituric acid reactive substances (TBARS) [7] and level of oxidized proteins, expressed as plasma carbonyls [12].

### 2.5. Monocyte Activation Parameters

In order to evaluate basal activation of circulating monocytes, cell surface HLA-DR and CD-11b levels were assessed by flow cytometry, as described elsewhere [13]. Monocytes were identified by monoclonal antibodies anti-CD14-pernidin chlorophyll protein (PerCP); neutrophils were excluded with CD66b-fluorescein isothiocyanate (FITC) labeling. Cell surface molecules were marked with anti-HLA-DR-phycoerythrin (PE) and anti-CD11b-allophycocyanin (APC); controls were incubated with anti-HLA-DR-PE and CD11b-APC isotypes.

Intracellular production of IL-1*α*, IL-6, and TNF-*α* was quantified by flow cytometry in monocytes stimulated with LPS and treated with monensin in order to prevent cytokine release [13]. Monocytes were identified by monoclonal antibodies for surface staining (anti-CD66b-FITC and anti-CD14-PerCP) and cytokine production was detected using monoclonal antibodies anti-IL-1*α*-PE, anti-IL-6-PE, and anti-TNF*α*-APC or their respective isotype controls.

Data were acquired using a FACSCalibur flow cytometer and CellQuest software (both from BD Immunocytometry Systems).

### 2.6. Reactive Oxygen Species Assay

Whole blood samples were first stained with anti-CD14 PerCP for monocyte identification; samples were subsequently incubated with 2′,7′-dichlorofluorescein diacetate (DCFH-DA), a stable compound, nonfluorescent, which diffuses into the cells and is hydrolyzed to 2′,7′-dichlorofluorescein (DCFH). Upon cell stimulation with LPS, DCFH was oxidized to DCF by reactive species, mostly H_2_O_2_, emitting high levels of green fluorescence, which was detected by flow cytometry [14] using a FACSCalibur flow cytometer and CellQuest software (both from BD Immunocytometry Systems).

### 2.7. Statistical Analysis

Normality of data distribution was assessed by Shapiro-Wilk test. Comparison between average results of the groups was performed using Student *t* test for independent samples. Correlation analysis between data was performed using Spearman correlation test. All data were processed and analyzed by appropriate statistical tests using SPSS 17 software.

## 3. Results

No changes were observed on plasma activity of the major erythrocyte antioxidant enzymes SOD, catalase, and glutathione peroxidase (Table 1). Table 1 also shows that total glutathione levels were similar in both cognitively intact (INT) and in group of patients with probable AD.

Lipid peroxidation (measured as TBARS) and plasmatic protein oxidation measured as the amount of circulating protein carbonyls did not significantly differ between the groups INT and AD (Table 2).

Plasma vitamin C levels were equivalent in both groups, as illustrated in Table 2. Values of circulating *α*-tocopherol were about 20% lower in patients with probable Alzheimer's disease compared with cognitively intact patients. This difference was statistically significant. It was observed that the two groups studied had similar plasma levels of *β*-carotene, lycopene, and coenzyme Q10 (Table 2).

Table 3 shows that Cu, Zn-SOD activity had a positive correlation with catalase and glutathione peroxidase activities, as well as total glutathione content. Catalase activity, in turn, also had positive correlation with glutathione peroxidase activity and total glutathione content in erythrocytes. Glutathione peroxidase activity and total glutathione content in red blood cells also had a significant positive correlation. The same two parameters negatively correlated with lipid peroxidation products in plasma.

**Figure 1:**
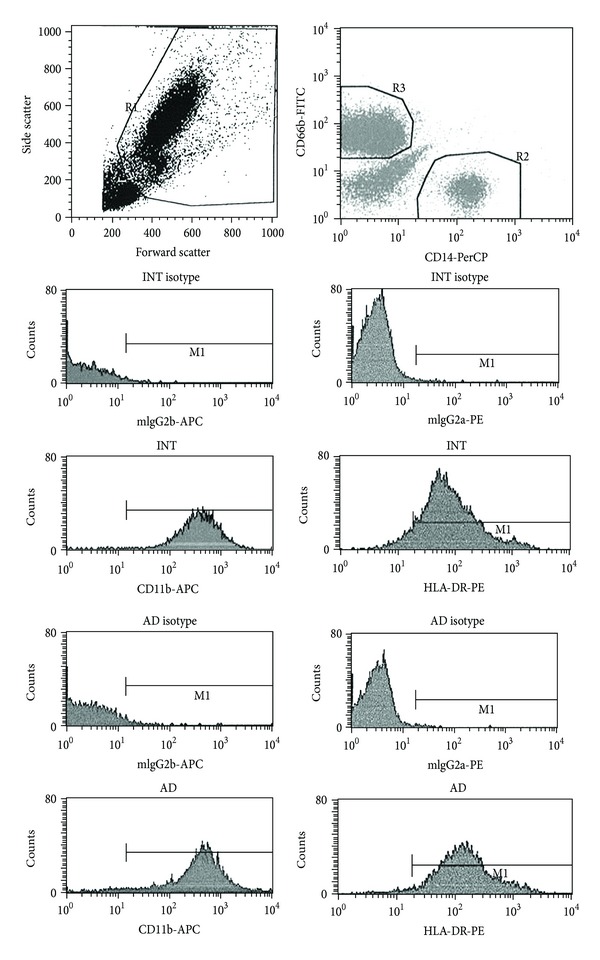
Representative flow cytometric analysis of whole blood samples stained with anti-CD11b-APC (right column) and anti-HLA-DR-PE (left column) monoclonal antibodies. From all events acquired in gate R1 (upper left graph), CD14-positive monocytes (gate R2, separated from CD66b-positive neutrophils in gate R3; upper right graph) were used for histogram calculation of the geometric mean fluorescence intensity (GMFI). A similar analysis was performed for the detection of fluorescent DCF in the ROS assay (histogram not shown). INT (sample from cognitively intact patient) and AD (sample from patient with probable Alzheimer's disease).

Monocytes of patients with probable Alzheimer's disease expressed approximately 70% more HLA-DR than the cognitively intact patients. On the other hand, there were no differences on the expression of CD11b by monocytes obtained from the two groups (Figure 1 and Table 4).

**Figure 2:**
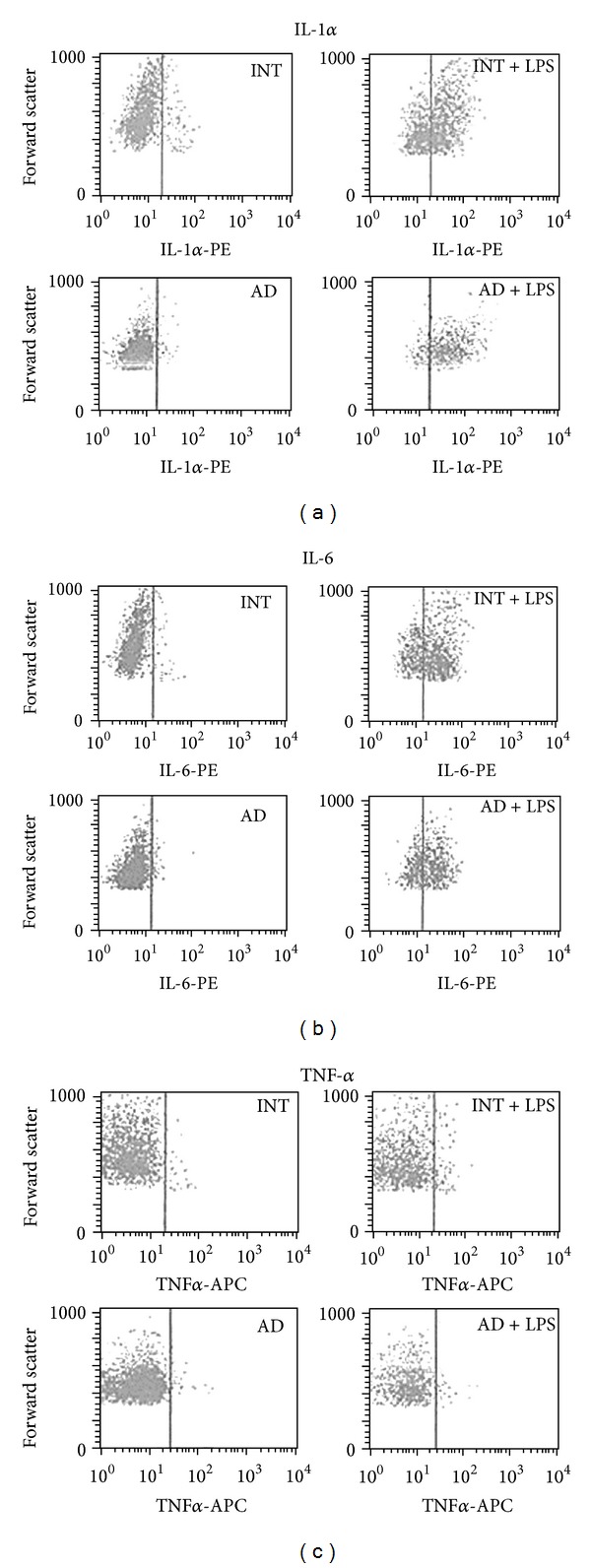
Representative flow cytometric detection of monocyte intracellular production of IL-1*α*, IL-6, and TNF-*α*. Monocytes were selected in a similar manner as described in Figure 1. LPS-induced production of each cytokine was determined by the percentage of events in the upper right quadrant (%URQ). Only upper quadrants are shown. INT (sample from cognitively intact patient) and AD (sample from patient with probable Alzheimer's disease).

The proportion of cells producing IL-1*α* was approximately 25% higher in patients with probable diagnosis of the disease as compared to those cognitively intact (Table 4). In the case of IL-6 and TNF-*α*, there were no statistically significant differences in the proportion of cells that produced those interleukins (Figure 2 and Table 4).

The oxidative metabolism of monocytes studied with flow cytometry techniques showed no differences between groups (Table 4).

## 4. Discussion

The aim of this study was to investigate the interrelationship between AD, blood parameters of oxidative stress, and indicators of inflammatory activity in monocytes. It follows the reasoning by which monocytes are circulating cells that resemble the brain microglia and may even be recruited from blood to brain tissue [15] in AD. Microglia are considered the main element for the localized inflammatory events that accompany this disease [16]. This would lead to localized inflammation and increased production of reactive oxygen species, causing oxidative stress, which could be reflected by circulating parameters [17].

In the conditions of this work, AD is not associated with any evidence of systemic oxidative stress. Although the activities of erythrocyte antioxidant enzymes do not show changes in patients with AD, Spearman rank correlation test reveals a positive correlation between the mutual activities of erythrocyte superoxide dismutase, catalase, and glutathione peroxidase and erythrocyte total glutathione levels. These data confirm the validity of the postulate that says that the body's antioxidant systems act together in an integrated manner. Spearman test has also identified a negative correlation between the levels of lipid peroxidation products, plasma glutathione peroxidase activity, and total glutathione levels in erythrocytes. This fact indicates that lipid peroxidation is influenced by the activity of antioxidant glutathione peroxidase-glutathione system in this experimental model. Indeed, glutathione-glutathione peroxidase system is an important component of antioxidant protection network to cells [18]. As a counterpoint to the measurement of erythrocyte antioxidant activities, circulating levels of oxidation products of macromolecules in the body have been evaluated. To this end, we analyzed levels of plasma protein carbonyls and plasma levels of lipid peroxidation products. Plasma level of protein oxidation products is not altered in AD compared with cognitively intact group. This lack of change is found in some studies [18]. On the other hand, some authors have found an increase of oxidized proteins in plasma from AD patients [19, 20]. This difference may be due to difference of sensitivity of the methods employed in the mentioned studies (two-dimensional electrophoresis, mass spectrometry) in relation to enzyme-immunoassay method chosen for this work and the possible differences in quality of care and feeding among the target populations of each study. Concentration of circulating lipid peroxidation products is equivalent in both groups, results that agree with those commonly found in the literature [18]. In general, the population studied seems to enjoy a privileged status in relation to nutritional populations examined in similar studies in other locations. In addition to the antioxidant profile outlined above, measurements of plasma levels of nonenzymatic antioxidants of low molecular weight were also carried out.

Values of vitamin C, *β*-carotene, and *α*-tocopherol, obtained in this study are within the range previously described for a healthy population group in that age [7, 21]. A review of several published papers, performed by Stocker and Frei [22], shows populational values for plasma concentrations of vitamin C, *α*-tocopherol, and *β*-carotene ranging from 30 to 150 *μ*M, 15 to 40 *μ*M, and 0.3 to 0.6 *μ*M, respectively. Other authors report that, for cardiovascular disease and cancer prevention, concentrations above 30 *μ*M *α*-tocopherol in combination with concentrations greater than 50 *μ*M of vitamin C, 0.4 *μ*M *β*-carotene, and 0.5 *μ*M lycopene are desired [23]. Thus, the two groups of elderly patients studied have positioned themselves within the limits found in several other studies to molecular plasma levels of antioxidants. These parameters reflect part of the antioxidant defense system of the body of patients, evaluating components that can be absorbed directly from the diet, so further promoted by the antioxidant defense mentioned above. Of all low molecular weight antioxidants studied, only vitamin E significantly differs between groups. The INT group has levels of plasma vitamin E higher than those in AD patients.

This apparent homogeneity in antioxidant levels between the various groups opposed to other studies in the literature [24–27]. This finding, as previously mentioned, reinforces the notion that the oxidative processes that are triggered during the course of AD are not always reflected in circulating blood, and even when that occurs, it may be only an indication of other underlying mechanisms. Of special interest is the fact that the establishment of AD, expressed by the comparison between the INT and AD groups in our study, is accompanied by the decrease of circulating vitamin E, which possesses antioxidant capabilities as well as recognized anti-inflammatory activities due to its action on signal transduction mechanisms in phagocytes [28, 29]. Not surprisingly, studies that have obtained the best results in preventing or treating AD in recent years used *α*-tocopherol as an agent [30].

To complete the picture outlined up to this point, circulating monocytes were analyzed by flow cytometry as indicators of inflammatory activity involved in AD. To this end, we quantified the level of expression of surface molecules HLA-DR, as an indicator of activation of circulating monocytes, and CD-11b, an adhesion molecule involved in the process of marginalization and migration of monocytes. The results show that AD patients have circulating monocytes that express higher amounts of HLA-DR on the surface even without external stimulus, indicating a higher basal activation state of these cells, confirming the data available in the literature [31]. Markers of increased activation of monocytes in the bloodstream are found in patients from a number of neurodegenerative diseases [32]. As previously mentioned, these circulating monocytes can cross the blood-brain barrier and migrate to the brain tissue, differentiating into microglia. Since circulating monocytes of AD patients are more active at baseline than those of INT, it can be postulated that microglia derived from these cells react more intensely to inflammatory stimuli, producing greater injury to brain. Level of expression of surface protein CD-11b is the same in both groups indicating that the potential for adhesion and migration of these cells is not altered in this pathology.

Also by flow cytometry, monocytes production of IL-1*α*, IL-6, and TNF-*α* was measured. This measurement was intended to evaluate the capacity of circulating monocytes to stimulate the inflammatory process by attracting new monocytes to the inflammatory site. The production of IL-1*α* is higher in monocytes from AD patients than in INT group. Since IL-1*α* is an important proinflammatory cytokine, it is plausible to assume that migration and differentiation of these monocytes produce more active microglia. This increased production of IL-1*α* was shown *in vitro* in human monocytes lineage, when stimulated by P*β*A, by means of mechanisms of signal transduction mediated by tyrosine kinases, which reinforces the above hypothesis [33].

Levels of IL-6 produced by stimulated peripheral monocytes are similar in both groups, as well as of TNF-*α*, agreeing with Beloosesky et al. [34]. Other authors detected an increase in IL-6 production by monocytes of AD patients [35] using, however, a different stimulus (phytohemagglutinin) than that used in this present study (LPS).

From the data shown here, it is reasonable to conclude that AD is accompanied by the activation of circulating monocytes and a decrease in circulating levels of vitamin E. Several studies show *α*-tocopherol modulating inflammation activity [28, 36]. In our study, the group of patients, where plasma *α*-tocopherol is decreased, also showed more activated monocytes that respond to stimuli with an increased production of proinflammatory cytokine IL-1*α*. This study points out that AD is associated with a higher basal activation of circulating monocytes that may be a result of *α*-tocopherol stock depletion.

## Figures and Tables

**Table 1:** Oxidative stress parameters in erythrocyte (RBC). Antioxidant enzymes: superoxide dismutase (SOD), catalase (CAT), and glutathione peroxidase (GPx), and total glutathione level (TGSH). INT (cognitively intact patients; *n* = 42) and AD (patients with probable Alzheimer's disease; *n* = 23). Values shown correspond to the means ± SEM. Comparison between average results of the groups was performed using Student's *t*-test for independent samples.

Parameter	INT	AD
RBC SOD (U/mg Hb)	7.82 ± 0.24	8.2 ± 0.31
RBC CAT (U/mg Hb)	187 ± 7	184 ± 8
RBC GPx (U/mg Hb)	12.8 ± 0.4	12.6 ± 0.7
RBC TGSH (mmol/Hb)	7.67 ± 0.21	7.45 ± 0.36

*P* < 0.05 was considered significant.

**Table 2:** Oxidative stress parameters in plasma. INT (cognitively intact patients; *n* = 42) and AD (patients with probable Alzheimer's disease; *n* = 23). Values shown correspond to the means ± SEM. Comparison between average results of the groups was performed using Student's *t*-test for independent samples.

Parameter	INT	AD
Vitamin C (*µ*M)	48 ± 3	52 ± 5
*α*-Tocopherol (*µ*M)	21 ± 1	17 ± 1*
*β*-Carotene (*µ*M)	0.79 ± 0.9	0.76 ± 1
Lycopene (*µ*M)	0.73 ± 0.09	0.67 ± 0.13
Coenzyme Q10 (*µ*M)	0.15 ± 0.02	0.13 ± 0.03
TBARS (thiobarbituric acid reactive substances) (nmol/mL)	5.82 ± 0.51	5.83 ± 0.62
Oxidized proteins (nmol/mg prot)	0.14 ± 0.01	0.16 ± 0.02

*P < 0.05 was considered significant.

**Table 3:** Correlation between antioxidant erythrocytes parameters and plasmatic oxidation products. Superoxide dismutase (SOD), catalase (CAT), glutathione peroxidase (GPx), total glutathione level (TGSH), and thiobarbituric acid reactive substances (TBARS). Analysis was performed using the Spearman correlation test.

Spearman test	SOD	CAT	GPX	GSH	TBARS
SOD					
Coefficient of correlation	1.000	0.415*	0.316*	0.406**	0.097
Significance		0.001	0.011	0.001	0.244
CAT					
Coefficient of correlation	0.415*	1.000	0.368**	0.317*	0.158
Significance	0.001		0.003	0.010	0.129
GPX					
Coefficient of correlation	0.316*	0.368**	1.000	0.416**	0.323**
Significance	0.011	0.003		0.001	0.009
TGSH					
Coefficient of correlation	0.406**	0.317*	0.416**	1.000	0.253*
Significance	0.001	0.010	0.001		0.034
TBARS					
Coefficient of correlation	0.097	0.158	0.323**	0.253*	1.000
Significance	0.244	0.129	0.009	0.034	

**P < 0.01 *, *P* < 0.05.

**Table 4:** Monocyte activation parameters. INT (cognitively intact patients; *n* = 42) and AD (patients with probable Alzheimer's disease; *n* = 23); GMFI: geometric mean fluorescence intensity; % URQ: percentage of events in the upper right quadrant. Values shown correspond to the means ± SEM. Comparison between average results of the groups was performed using Student *t* test for independent samples.

Parameter	INT	AD
HLADR (GMFI)	109.93 ± 10.59	177.74 ± 30.64*
CD11B (GMFI)	406.74 ± 39.24	412.92 ± 48.97
IL-1*α* (%URQ)	65.78 ± 2.46	82.82 ± 6.52*
IL-6 (%URQ)	52.89 ± 4.31	49.71 ± 6.57
TNF-*α* (%URQ)	6.85 ± 1.28	8.26 ± 2.47
Oxidative metabolism (GMFI)	266.32 ± 38.99	257.77 ± 63.07

*P < 0.05 was considered significant.

## Abbreviations

AD: Alzheimer's disease
APC: Allophycocyanin
CDR: Clinical dementia rating
FITC: Fluorescein isothiocyanate
INT: Cognitively intact group
GMFI: Geometric mean fluorescence intensity
LPS: Lipopolysaccharide
MMSE: Minimental state examination
PE: Phycoerythrin
PerCP: Periidinin chlorophyll protein
ROS: Reactive oxygen species
URQ: Upper right quadrant.
